# Social Determinants and Poor Diet Quality of Energy-Dense Diets of Australian Young Adults

**DOI:** 10.3390/healthcare5040070

**Published:** 2017-10-01

**Authors:** Amanda Grech, Anna Rangan, Margaret Allman-Farinelli

**Affiliations:** Nutrition and Dietetics Group, The School of Life and Environmental Science, The Charles Perkins Centre D17, The University of Sydney, Sydney, NSW 2006, Australia; anna.rangan@sydney.edu.au (A.R.); maragaret.allman-farinell@sydney.edu.au (M.A.-F.)

**Keywords:** dietary energy density, diet quality, eating index, healthy, obesity, young adult

## Abstract

This research aimed to determine the diet quality and socio-demographic determinants by level of energy-density of diets of Australian young adults. Secondary analysis of the Australian National Nutrition and Physical Activity Survey-2011/2012 for adults aged 18–34 years (*n* = 2397) was conducted. Diet was assessed by 24-h recalls. Dietary energy-density was calculated as dietary energy/grams of food (kJ/g) and the Healthy-Eating-Index-for-Australians (HEIFA-2013) was used to assess diet quality (highest score = 100). Dietary energy-density was examined with respect to diet quality and sociodemographic determinants including gender, highest tertiary-education attainment, country-of-birth, age, income, and socio-economic-index-for-area (SEIFA). Higher dietary energy-density was associated with lower diet quality scores (β = −3.71, *t* (2394) = −29.29, *p* < 0.0001) and included fewer fruits and vegetables, and more discretionary foods. The mean dietary energy-density was 7.7 kJ/g and 7.2 kJ/g for men and women, respectively. Subpopulations most at risk of consuming high energy-dense diets included those with lower education, Australian and English-speaking countries of birth, and men with low income and women from areas of lower socio-economic status. Young adults reporting low energy-dense diets had higher quality diets. Intensive efforts are needed to reduce the high energy-density of young adults’ diets, and should ensure they include populations of lower socio-economic status.

## 1. Introduction

Young adults have been shown to be gaining weight faster than other age groups and each new generation is at greater risk of overweight and obesity than the previous [1,2,3]. As such, obesity rates are predicted to increase [3]. There is a large personal and economic cost of overweight and obesity due to increased risk of non-communicable disease, and interventions to prevent obesity are urgently required [4,5]. Low energy-dense diets can assist in preventing weight-gain [6,7,8] and the World Cancer Research Fund (WCRF) recommends that dietary energy-density should be less than 5.23 kJ/g [8]. However, there is limited research that has quantified the energy-density of young-adults’ diets or determined which subpopulations of young adults are most at risk of consuming high energy-dense diets.

Dietary energy-density (kJ/g) is an important determinant to the overall energy consumed [9,10]. Energy-density can lead to passive over-consumption, as people eat a fairly constant volume of food from day to day [9]. Therefore, the greater energy per gram of food consumed, the greater the total energy consumed [9]. Decreasing energy-density by consuming more low energy-dense foods has been shown to be effective in weight-loss interventions [11,12], while high energy-dense diets can lead to weight-gain [13,14]. In Australia, the prevalence of overweight and obesity in 2014–2015 was 45% for men and 34% for women aged 18–24 years increasing to 62% for men and 41% for women aged 25–34 years old [15]. Obesity prevalence is higher for those with lower educational attainment and for women from socio-economically disadvantaged areas [16]. High dietary energy-density is a plausible reason why lower socio-economic status (SES) is correlated with a higher prevalence of obesity, as such diets have been shown to be associated with lower diet costs in the United States and France [17,18]. However, there is no known analysis of the dietary energy-density for populations of different socio-economic backgrounds in the Australian population or for young adults.

Energy-density increases with fat content and is lowered by the water content of foods. Foods higher in energy-density can include discretionary foods such as fast foods, snack foods, cakes, and biscuits [9,10] but can also include healthier, nutrient dense foods, such as breakfast cereals, olive oil, nuts, cheese, and bread. Similarly, nutrient poor foods that contain higher levels of deleterious nutrients to health, such as sodium and added sugars [5], can be lower in energy-density, for example, sauces and ice-cream. It therefore cannot be assumed that low energy-dense diets are necessarily better quality. While some evidence suggests that low energy-dense diets are of higher quality [19,20], this has yet to be established in the Australian population and it is unknown if those with lower energy-dense diets more closely adhere to the Australian dietary guidelines.

The aim of this research is two-fold: to determine if low energy-dense diets of young adults are of better diet quality and more closely adhere to the Australian Dietary Guidelines; and to determine if there is a relationship between high energy-dense diets and socio-demographic background.

## 2. Materials and Methods 

The National Nutrition and Physical Activity Survey-2011/2012 (NNPAS-2011/2012) was conducted by the Australian Bureau of Statistics (ABS). The survey was a cross-sectional multi-staged area sample of 97% of the Australian population and was designed to provide sample sizes sufficient for analysis by age-groups and sex for the total population. Extensive details on the methodology are published elsewhere [21]. The survey was conducted under the Federal Census Act 1905. This work involved secondary analysis of de-identified data and was exempt from full review from the Institutional Review Board.

### 2.1. Dietary Assessment

Twenty-four-hour diet recalls were collected from May 2011 through to June 2012. The five-pass ‘Automated Multiple-Pass Method’ developed by the United States Department of Agriculture was used, and had been modified with assistance from Food Standards Australia and New Zealand (FSANZ) to reflect the Australian food supply. All participants were invited to participate in a second recall, however, participation rates declined and included only 60.8% of participants from the first survey. Only day one data was used for this analysis, but a single recall is sufficient to provide estimates of group means [22]. Day one interview was conducted with a computer-assisted-personal-interview (CAPI). Interview days included Monday through to Sunday. The interviews were conducted by highly trained technicians selected from the ABS panel. The food composition database “AUSNUT-2013” was constructed by FSANZ specifically for the purpose of assessing the nutrient composition of the foods reported in the NNPAS-2011/2012 and therefore reflects the food supply at the time of the survey [23].

### 2.2. Dietary Energy-Density

Dietary energy-density was calculated as sum of daily kilojoules from food/sum of the total grams of food (kJ/g) reported on the day of the survey. Beverages were excluded from the energy-density calculation, as beverages have relatively low energy-density compared to food and as such can obscure the relationships between exposure to energy-dense foods and health outcomes [24,25]. Consistently, the WCRF have made recommendations that dietary energy-density should be less than 5.23 kJ/g, which was calculated for food only, and have separate guidelines for beverages [8]. Milk incorporated into food was included in the calculation for energy-density (e.g., milk on cereal), but all milk-based beverages were excluded. Beverage intake was considered separately in this analysis, as described below.

### 2.3. Diet Quality

Diet quality was assessed with a validated diet quality index, the Healthy-Eating-Index-for-Australians-2013 (HEIFA-2013) [26]. Full details of the validation and scoring system are published elsewhere [26,27,28]. The tool assesses compliance to the Australian Dietary Guidelines–2013 (ADG) and consists of ten components which assess intake of the (1) serves of discretionary food; (2) serves of vegetables and variety of vegetables (orange, green and brassica, starchy, legumes and tubes and bulbs); (3) serves of fruit and variety of fruit (citrus, berry, pome, tropical, stone fruit, and other); (4) serves of dairy products and alternatives (i.e., milk, yoghurt, cheese, and non-dairy alternatives such as soy milk); (5) serves of meat, poultry, and alternatives; (6) serves of grain foods and wholegrains; (7) percentage energy from saturated fat and serves of polyunsaturated fat; (8) sodium; (9) percentage of energy from added sugars; (10) water and alcohol. Each component can achieve a maximum of 10 marks. Scores were given incrementally based on how closely ADG recommendations were met, i.e., maximum scores were given for meeting recommendations and no scores were given for consuming less than a minimum threshold. Criteria for maximum scores for nutrients were: <10% of energy from saturated fat; <5% of energy from added sugars; >50% of fluids from water; and <20 g of alcohol per day [26,28]. The serve sizes and recommended number of serves of foods required to receive the maximum HEIFA-2013 scores are shown in Table 1.

The number of serves of the foods reported by each participant were supplied by the Australian Bureau of Statistics. Each recipe in the AUSNUT database was disaggregated into its component parts so that the reported serves reflect the actual reported intake of each food group. For example, lasagna was disaggregated into its components: vegetables (e.g., tomato, onion), grains (pasta), and dairy products (cheese and milk). Discretionary foods are defined as those that are high in added sugar (e.g., sugar-sweetened beverages, syrups), saturated fat (e.g., pastry, butter, crisps, and pizza or burgers with more than 5 g of saturated fat per serve), added sodium (sauces and gravies), or alcohol (e.g., wine, beer, and spirits). These foods were not included in the calculation of the number of serves of other food groups. For example, milk in milk chocolate was only included in the discretionary food group and did not contribute to the ‘milk, yoghurt, cheese, and alternatives’ group. A full list of the 1630 foods (28.8%) classified as discretionary foods in the AUSNUT database is available on the ABS website [21].

### 2.4. Implausible Energy Reporting

Participants with an implausible energy intake were identified as those with energy intake: basal metabolic rate ratio (EI: BMR) of < 0.87 [29]. Participants were categorized as either low-energy reporter (EI: BMR < 0.87) (*n* = 167, 16.3% males and *n* = 235, 22.1% females) or as plausible energy reporter (EI: BMR ≥ 0.87) or unknown. This was included as a co-variate and reported means are adjusted for the effects of low energy reporting.

### 2.5. Statistical Analysis

Differences in diet were examined in three ways. (1) Change in diet quality scores, measured with the HEIFA-2013, with increasing dietary energy-density; (2) Intake of food groups (i.e., serves of vegetables, fruit, grain foods, meat, poultry, and alternatives, dairy products and alternatives, discretionary food and beverages, and polyunsaturated fat) for those reporting dietary energy-density ≤5.23 kJ/g in line with the WCRF recommendations compared to those exceeding the WCRF recommendation with dietary energy-density >5.23 kJ/g. Serve sizes of food groups are presented in Table 1. (3) The amount of foods consumed for ‘sub-major’ food groups (e.g., burgers, potatoes, or chocolates) for participants with dietary energy-density of ≤5.8 kJ/g, 5.8–8.9 kJ/g, and ≥8.9 kJ/g, for foods reported by >10% of young adults. Sub-major food groups were categorized by the ABS. The energy-density of each sub-major food group was also calculated as the sum of the energy (kJ) of food reported by young adults in each sub-major food group/sum of the grams of the food in each sub-major food group. Differences in food group intake and sub-major food group intake for people with different dietary energy-density were assessed with a generalized-linear-model (GLM) in SAS proc GLM and adjusted for energy-reporting status.

The socio-demographic determinants of dietary energy-density were examined for the 2011/2012 population of young adults. Socio-demographics for analysis included country of birth: Australia, other main English-speaking countries (Canada, Ireland, NZ, South Africa, UK, USA), and all other countries; age-groups 18–24, 25–29, and 30–34 year olds; level of education: Bachelor or higher, technical colleges/vocational, no tertiary education, and studying at time of interview (level of study was not provided); equalized household income expressed in quintiles; and socio-economic index for area (SEIFA) created by the ABS, a relative index for socio-economic-status (SES) expressed as quintiles [30]. Analyses were conducted for the total population and for males and females separately.

Linear regression was used to assess change in diet quality scores with increasing dietary energy density. ANOVA was used to test for significant mean differences between groups and adjusted for age and low energy reporting status. A multiple regression model was used to estimate the adjusted contribution of each significant socio-demographic variable. Analyses were generated using SAS software, Version 9.4 for Windows. Copyright © 2002-2012 SAS Institute Inc., Cary, NC, USA.

## 3. Results

Response rates for the NNPAS-2011/2012 was 77% of persons contacted [21]. The full NNPAS-2011/2012 sample included *n* = 12,153 persons aged >2 years. A total of 2397 participants aged 18–34 years (53.27% females) were included in this analysis.

**Figure 1:**
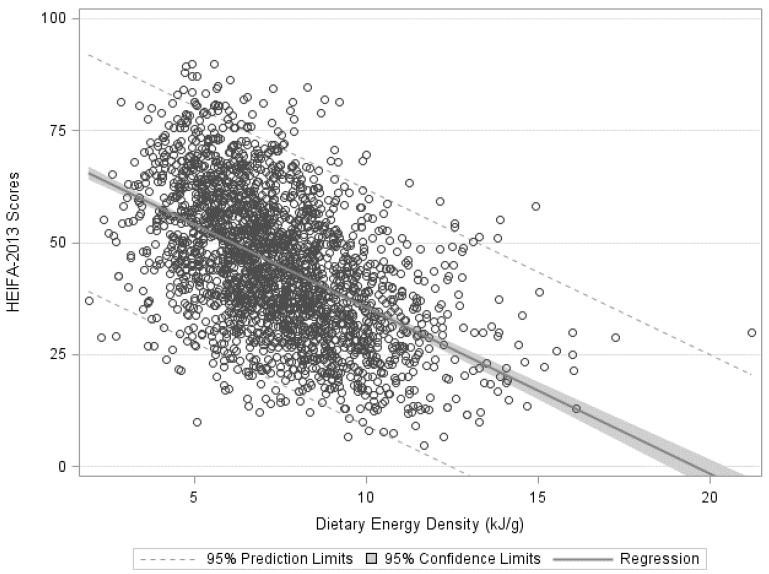
Diet quality of young Australian adults as measured with the Healthy-Eating-Index-for-Australian’s (HEIFA-2013) for different dietary energy-density (kJ/g).

**Figure 2:**
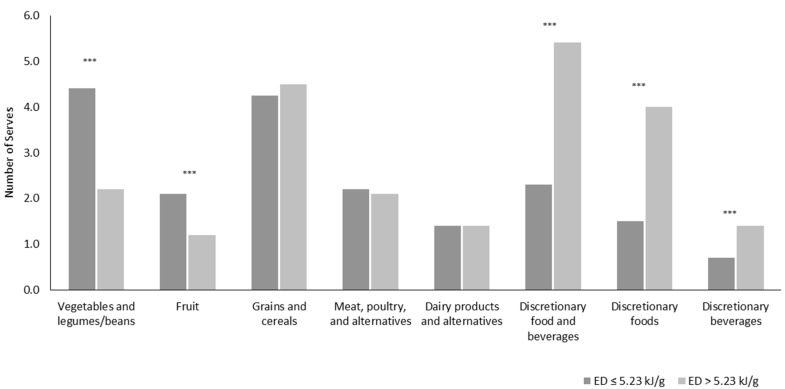
Mean food group intake for Australian young adults (aged 18–34 years) reporting low dietary energy-density (≤5.23 kJ/g) compared to those reporting higher dietary energy-density (>5.23 kJ/g). One serve = 75 g of vegetables; 125 mL of juice, 150 g of fresh or 30 g dried fruit; 500 kJ of grains and cereals; 500–600 kJ of meat, poultry, and alternatives; 500–600 kJ of milk, yoghurt, cheese, and alternatives; 7 g of polyunsaturated oil or 10 g of polyunsaturated spread/nuts/nut spread and 600 kJ of discretionary foods. *** *p <* 0.001. Means are adjusted for low-energy reporters (energy intake: basal metabolic rate ratio of <0.87). Survey-specific weighting factors were applied.

Diet quality scores explained a significant amount of the variance of dietary energy-density (F (1, 2394) = 858.08, *p* < 0.0001, *R*^2^ = 0.26) and diet-quality was poorer with increasing dietary energy-density (β = −3.71, *t* (2394) = −29.29, *p* < 0.0001) (Figure 1). There was little change in these results when adjusted for age, energy misreporting status, and SEIFA (β = −3.64, *t* (2394) = −28.46, *p* < 0.0001). Figure 2 shows the mean intake of food groups for those reporting low energy-dense diets in line with the WCRF (≤5.23 kJ/g) compared to those reporting higher dietary energy-density (>5.23 kJ/g). The mean number of serves of vegetables and fruit for those with low-energy-dense diets was higher compared to those that reported higher energy-density on the day of the survey at 4.4 serves and 2.2 serves for vegetables and 2.1 and 1.2 serves of fruit, respectively (*p <* 0.001). Intakes of meat and alternatives, dairy products and alternatives, and polyunsaturated fats did not differ. Discretionary food and discretionary beverage intake was also lower at 1.5 serves of discretionary food and 0.7 serves of discretionary beverages for those with low energy-dense diets compared to 4.0 serves and 1.4 serves of discretionary beverages for those with diets of higher energy-density (*p <* 0.001).

The energy-density of all foods consumed by more than 10% of young adults is shown in Table 2. The majority of energy-dense foods reported were discretionary foods including sugar, sweet biscuits, fried potatoes, chocolate, pastries, cakes, and butter. Consumption of most energy-dense core foods such as bread and cheese demonstrated a positive relationship with overall dietary energy-density, but breakfast cereal was an exception and had higher consumption for those with lower energy-density. Core foods of moderate energy-density such as poultry, beef, sheep, and pork dishes, and eggs showed no difference between tertiles of energy-density. Foods typically lower in energy-density were associated with lower dietary energy-density.

The mean (SD) dietary energy-density was 7.67 kJ/g and 7.24 kJ/g for males and females, respectively, and 7.40 kJ/g for all young adults. The dietary energy-densities for subpopulations of young adults are shown in Table 3. Participants aged 18–24 years had higher energy-density than those aged 30–34 years at 7.38 kJ/g and 7.13 kJ/g, respectively (*p* = 0.04). Young adults born in Australia and other English-speaking countries had higher mean dietary energy-density at 7.36 kJ/g than those born in non-English-speaking countries at 6.77 kJ/g (*p <* 0.0001). Women from areas of socio-economically disadvantage (i.e., SEIFA quintile 1) had dietary energy-density of 7.40 kJ/g which was significantly higher than those from the most advantaged areas, which was 6.88 kJ/g. Young adults with university education had the lowest energy-density, with 6.85 kJ/g for those with a university qualification compared to 7.53 kJ/g for those without tertiary education (*p* <0.0001). Higher income was also associated with lower energy-density for men but not women. Differences between country of birth for men was not independently associated with energy-density (Table 3).

## 4. Discussion

High energy-dense diets of young Australian adults were of poor quality with the greatest disparity from dietary guidelines. Therefore, those with the most energy-dense diets are not only at greater risk of weight-gain, but also at risk of other health consequences of a poor quality diet, such as cardiovascular disease and stroke, some cancers, and diabetes [5]. Differences in diet quality for those with high and low energy-dense diets were due to higher intake of discretionary foods, which are high in added sugar, sodium, and saturated fat, and lower intake of fruit and vegetables. The average dietary energy-density of young adults’ diets exceeded recommendations of the WCRF for all young adults but was higher for those with lower educational attainment and women from areas of lower SES and men with lower income reported more energy-dense diets. Interventions that aim to lower dietary energy-density will need to address low fruit and vegetable intake and high intake of discretionary foods

The average dietary energy-density of young-adults was comparatively higher than reported in other countries. The energy-density of the diets of Japanese adults was 5.98 kJ/g for men and 5.72 kJ/g for women [31] and 5.2 kJ/g and 4.6 kJ/g for Spanish men and women, respectively [32]. However, it was lower than dietary energy-density of adults aged 50 years and under from the USA which was 8.5 kJ/g [33]. Correspondingly, the prevalence of obesity is also lower in both Japan and Spain, and higher in the USA than it is in Australia [34]. The average dietary energy-density of Iranian young women ranged between quartiles of low to high dietary energy-density, from 5.3 kJ/g–7.1 kJ/g, respectively, and the prevalence of overweight and obesity ranged from 8–30% [20]. However, estimates for dietary energy-density for Spanish and Iranian populations were derived with food-frequency-questionnaires [20,32] and therefore do not directly compare to the estimates derived from studies using 24-h recalls, including this study. As increasing dietary energy-density has been shown to increase overall energy intake [9], young adults are on average consuming diets higher in energy and consequently are at risk of perturbing energy-balance in favour of weight-gain. Indeed, much of the Australian population will become overweight in their lifetime and it is projected that more than 70% of the Australian adult population will be overweight by 2025 [3], an increase from 62.8% in 2012 [35]. Weight-gain during early and middle adulthood (ages 18–35 and 35–50 years, respectively) elevates mortality, with weight gain during young adulthood demonstrating the strongest relationship with mortality [36]. The lifestyle choices that young adults make clearly have serious consequences for their future health. The efficacy of reducing dietary energy-density of young adults to modify this trajectory should be examined.

Consistent with findings internationally [32,37,38,39], diet quality was higher for young adults with lower dietary energy-density. Similar to the present analysis, assessment of the diet of young adult women from Iran demonstrated that low dietary energy-density was associated with higher Healthy Eating Index scores [20]. Higher diet quality assessed with diet quality indices has been demonstrated to be associated with reduced all-cause mortality, cardiovascular disease, and cancer [40]. By extension, those consuming low energy-dense diets are at lower risk of chronic disease. Improved diet quality is most frequently found to be related to higher intakes of fruit and vegetables and lower intake of discretionary foods [32,37,38,39]. For example, in Irish adults differences in dietary energy-density were mostly due to variation in fruit and vegetables and sugar-sweetened beverages and younger adults consumed the most energy-dense diets [37]. In the present analysis it was found that intakes of meat, poultry, and alternatives, dairy products, and grains either did not change or were slightly higher for those with lower dietary energy-density. This implies that it is the combination of increasing fruit and vegetable intake and decreasing consumption of discretionary foods that will be most effective at lowering dietary energy-density for the young-adult population. Consistently, findings from a weight-loss intervention in young adults found that the small persistent behaviour change of increasing vegetable intake mediated weight-loss [41]. Energy-density increases with energy derived from fat [11], however, low energy-dense diets reported by free-living populations here and elsewhere are within the acceptable macro-nutrient distribution range for fat intake and/or have no difference in beneficial polyunsaturated fats but are overall lower in saturated fat [20,42]. 

Although internationally disparities in SES are commonly found for numerous health behaviours, there is still no consensus as to why these disparities exist [43]. Studies in the US and France proposed that a higher prevalence of obesity for those of lower SES may be due to the greater expense of lower energy-dense diets and higher dietary energy-density [19,44]. Lower income was related to higher dietary energy-density for men but not women. Food insecurity is a concern for approximately 4% of Australians and in the past young adults have been shown to be a high risk group with estimates as high as 15% at risk of food insecurity [45], although these figures have not been updated in recent years. Income, however, is only one potential aspect of health inequality and other barriers identified include differences in social support and influence, lack of community opportunity, and/or limited access to healthy foods [43]. Higher educational attainment is also an important aspect of socio-economic inequality and was inversely associated with dietary energy-density for both men and women. Increasing education is associated with improved self-efficacy and agency and is therefore thought to improve the sense of control to partake in activities that are beneficial for health, such as choosing to consume more vegetables [43].

Many of the barriers identified for those of lower socio-economic status are also named by young adults, such as limited confidence and limited access to healthy options, while having friend and family role models participating in healthy behaviours improved their behaviour [46,47,48]. Cost is also a common barrier named by young adults to consuming healthier diets [46,48,49]. It has been demonstrated that diets consistent with the dietary guidelines cost less to purchase than the population’s current expenditure on food [50] and it is the perception that healthy diets are more expensive rather than the real cost [50]. Evidence from a successful randomized-controlled health intervention in young adults demonstrated that socio-demographic factors including education and SEIFA did not alter weight-loss during the intervention, while income moderated the outcome at 3 months for men, but this was no longer true at 9 months, and therefore the program was suitable for most young adults [41].

The calculation for dietary energy-density in the present analysis did not include beverages, as the validity of including beverages in the energy-density calculation has been disputed in a systematic review of energy-density [24]. Inclusion of beverages demonstrably reduces dietary energy-density because of the higher weight of beverages compared to foods, and this can distort true associations between exposure to energy-dense foods and health outcomes [24]. However, of all adults, intake of sweetened beverages is the highest for 19–30 year olds [51] and should be considered in addition to energy-dense foods. It was demonstrated here that those reporting higher dietary energy-density also had higher intake of sweetened beverages. Therefore, interventions that target those with higher dietary energy-density are likely to also capture young adults with the highest intake of sweetened beverages.

Although the multiple pass 24-h recalls are a valid and reliable tool for dietary assessment, measurement error is fundamental in self-reported data due to difficulty in estimating portion sizes, nutrient composition of foods, or difficulty for the participant in recalling foods consumed [52]. Every effort was made to minimize error in the NNPAS-2011/2012, with vigorous methodology such as the use of life-size food models to assist participants in estimating portion sizes and a database containing information for some 15,847 measures of different food (e.g., packet size of available processed foods), a nutrient database that was made specifically for nutrient analysis of the foods reported in the survey, and use of the automated-multiple-pass methodology to assist participants in accurate recall [21]. However, low energy-reporting was still evident. The use of energy-density assists in correcting for error in self-reported dietary data because the error in energy is correlated to the error of all dietary components and as such nutrient intakes per 1000 kJ (or kJ/g used here) are more reliable than absolute values [52]. It is known that people tend to over-report intake of foods perceived to be more socially desirable such as vegetables, while they under-report discretionary foods, and dietary energy-density may be under-estimated here [53,54]. However, it is clear that dietary energy-density is high in the young-adult population and their diet-quality is low. This analysis is cross-sectional and offers a description of the energy-density of young adults’ diets but does not attempt to demonstrate causation; evidence is required to assess the long-term effects of low energy-dense diets and body-weight, and to determine the potential associated risks of other non-communicable diseases [55].

## 5. Conclusions

In conclusion, the reported dietary energy-density of Australian young adults was high and of poor diet quality. There was evidence of differences for people of lower tertiary education attainment and lower SES—this information should be used to help ensure that interventions include those most at risk of consuming higher dietary energy-density. Efforts to improve dietary patterns by increasing consumption of low energy-dense fruits and vegetables must be escalated while consumption of energy-dense, nutrient poor foods should be actively discouraged.

## Figures and Tables

**Table 1:** Serve sizes of foods and nutrients and recommended serves from the Australian Dietary Guidelines.

		ADG Recommended Serves	
Dietary Component	Serve size	Men	Women
Vegetables and legumes	75 g	6	5
Fruit	150 g ^1^	2	2
Grains	500 kJ	6	6
Wholegrains ^2^	500 kJ	>50% of grains	>50% of grains
Meat, poultry, and alternatives	500–600 kJ	3	2.5
Dairy products and alternatives	500–600 kJ	2.5	2.5
Discretionary food and beverages	600 kJ	0–3	0–2.5
Unsaturated fat (mono and poly)	7–10 g ^3^	4 serves	2 serves

ADG, Australian Dietary Guidelines. ^1.^ One serve of dried fruit = 30 g, 1 serve of fruit juice = 125 mL; ^2.^ The guidelines state “eat plenty of grain foods, mostly wholegrains” which has been interpreted as >50%; ^3.^ 7 g of unsaturated oil or 10 g of unsaturated spread or 10 g nut/nut spreads.

**Table 2:** Proportion of consumers (%), energy-density, and the amount consumed by those with dietary energy-density <5.8 kJ/g, 5.8–8.9 kJ/g, and >8.9 kJ/g for foods reported by Australian 18–34-year-olds on the first interview of the National Nutrition and Physical Activity Survey-2011/2012 (*n* = 2397).

					Mean Amount of Food Consumed (g)			
Food ED	Food	%	ED Mean	(SD)	DED < 5.8 kJ/g	DED 5.8–8.9 kJ/g	DED > 8.9 kJ/g	*P* for Trend
>9.41 kJ/g	Regular breads and bread rolls	56	11.1	(1.0)	40.7	55.4	59.22	<0.0001
	Sugar, honey, and syrups	42.1	15.4	(1.5)	6.3	8.2	8.1	0.06
	Cheese	30.9	14.5	(2.6)	8.9	13.2	13.5	0.0004
	Breakfast cereals, ready to eat	28.4	15.3	(1.0)	19.9	20.8	13.4	<0.001
	Margarine and table spreads	18.6	23.2	(3.5)	1.2	2.3	3.0	<0.0001
	Sweet biscuits	18	19.4	(1.8)	4.4	6.9	11.5	<0.0001
	Fried Potatoes	17.8	10.7	(1.8)	3.0	16.2	31.4	<0.0001
	Salad dressings	17.2	18.2	(10.3)	3.1	4.1	4.5	0.035
	Chocolates	17	20.9	(2.0)	3.8	8.7	12.7	<0.0001
	Pastries	14	10.8	(2.7)	14.0	26.2	42.3	<0.0001
	Nuts and nut products	13	25.3	(3.7)	3.6	5.8	4.4	0.08
	Cakes, muffins, and scones	12.9	14.7	(2.1)	7.8	18.3	29.8	<0.0001
	Savoury biscuits	12.6	18.3	(1.9)	2.6	4.4	6.2	0.003
	Other breads ^1^	12.3	12.2	(1.3)	10.8	10.9	10.8	0.9
	Butters	11.6	30.4	(1.1)	0.8	1.6	1.8	0.005
	Mixed dishes-grain based ^2^	45.5	8.3	(2.4)	132.7	171.4	167.5	0.002
	-Burgers	10.6	9.6	(1.2)	3.8	28.9	51.7	<0.0001
	-Pizza	9.6	10.8	(0.9)	4.9	15.8	45.0	<0.0001
>5.23–9.41 kJ/g	-Pasta dishes	16.8	5.6	(1.5)	72.2	68.7	18.3	<0.0001
	Poultry and feathered game	19.4	7.3	(2.3)	36.8	28.5	25.3	0.07
	Rice and other cereal grains ^3^	18.4	7.1	(1.9)	62.1	40.6	13.2	<0.0001
	Beef, sheep, and pork dishes	18.4	8.9	(3.3)	30.9	32.0	25.9	0.38
	Mixed poultry/game dishes ^4^	16.3	8.8	(2.7)	35.2	45.7	38.9	0.15
	Ice cream and frozen yoghurt	12.4	8.4	(1.9)	10.7	19.3	12.1	0.0006
	Mixed red meat dishes	11.3	6.6	(1.9)	30.0	38.6	16.3	0.0017
	Eggs	10.1	6.7	(1.3)	7.1	7.4	6.3	0.75
≤5.23 kJ/g	Gravies and savoury sauces	26.1	4.9	(3.6)	13.7	14.6	12.6	0.8
	Dairy milk	25.7	2.4	(0.8)	227	116.2	34.5	<0.0001
	Other fruiting vegetables	23.1	3.1	(2.9)	25.9	19.8	6.0	<0.0001
	Mixed vegetable dishes ^5^	21.8	3.4	(2.0)	52.3	30.5	21.3	<0.0001
	Pome fruit	21.6	2.4	(0.1)	73.3	33.8	9.4	<0.0001
	Leaf and stalk vegetables	21.2	0.8	(0.6)	8.5	6.7	3.8	0.0006
	Other vegetables	20.4	2.7	(2.1)	27.8	14.4	3.7	<0.0001
	Tomato and tomato products	18.6	1.2	(1.8)	15.1	10.7	6.2	0.0006
	Carrot and root vegetables	17.6	1.9	(1.3)	20.8	11.2	4.2	<0.0001
	Tropical and subtropical fruit	17.0	3.6	(0.6)	43.2	19.5	6.5	<0.0001
	Yoghurt	13.3	3.7	(1.1)	34.3	22.2	3.9	<0.0001
	Potatoes	12.1	4.1	(1.7)	18.1	20.5	4.6	<0.0001

ED, energy-density of foods. SD, standard deviation. DED, dietary energy-density of participants. ^1.^ English-style muffins, flat breads, and savoury/sweet breads ^2.^ Group comprised of pasta and noodle dishes (30%), pizza (16%), burgers (20%), sandwiches (11%), rice-based dishes (8.2%), tacos and tortilla dishes (7%), sushi (6%), other savoury grain dishes (1%) ^3.^ Group predominantly rice (92%) ^4.^ Group comprised predominantly of crumbed and battered chicken (55%) ^5.^ Group comprised of salads (85%) and dishes such as curries, stir-fries, and casseroles. Foods are grouped in accordance with the World Cancer Research Fund criteria for energy-density. Means are adjusted for low-energy reporters (energy intake: basal metabolic rate ratio of <0.87).

**Table 3:** Dietary energy-density (kJ/g) for subpopulations of young adults using dietary data from the first interview of the National Nutrition and Physical Activity Survey-2011/2012.

	Males		Females		Total	
Demographics and SES	n	Mean DED kJ/g	n	Mean DED kJ/g	n	Mean DED kJ/g
**Age**						
18–24	373	7.71	407	7.16a	780	**7.38a**
25–29	342	7.50	394	6.95a	736	**7.17b**
30–34	405	7.53	476	6.83b	881	**7.13b**
***p*-Value**	0.32		0.15		**0.046**	
**Country of birth**						
Australia or English Country	905	**7.68**	1070	**7.17**	1975	**7.36**
Other	215	**7.30**	207	**6.23**	422	**6.77**
***p*-Value**	**0.02#**		**<0.0001**		**<0.0001**	
**Income**						
Quintile 1-Lowest 20%	130	**7.76abc**	159	7.22ab	289	7.39a
Quintile 2	177	**8.12a**	160	6.97ab	337	7.42a
Quintile 3	187	**7.40bc**	172	6.91ab	359	7.08ab
Quintile 4	167	**7.56bc**	125	7.07a	292	7.25a
Quintile 5-Highest 20%	197	**7.40bc**	211	6.69b	408	6.98b
***p*-Value**	**0.01**		0.14		0.06	
**SEIFA**						
Quintile 1-Lowest 20%	130	7.84a	159	**7.40a**	289	**7.26a**
Quintile 2	177	7.60ab	160	**7.09ab**	337	**7.30ac**
Quintile 3	187	7.70ab	172	**6.92b**	359	**7.22bc**
Quintile 4	167	7.41b	125	**6.68b**	292	**7.00b**
Quintile 5-Highest 20%	197	7.42b	211	**6.88b**	408	**7.09bc**
***p*-Value**	0.18		**0.005**		**0.005**	
**Education**						
University	333	**7.33a**	364	**6.56a**	697	**6.85a**
Student (level not specified)	120	**7.69ab**	87	**6.86ab**	207	**7.21b**
Vocational	217	**7.59a**	207	**7.59c**	424	**7.51c**
None	188	**7.93b**	169	**7.15b**	357	**7.53c**
***p*-Value**	**0.004**		**<0.0001**		**<0.0001**	

SES, Socio-economic status. DED, Dietary energy-density (kJ/g). SEIFA, Socio-economic indexes for area developed by the ABS that ranks Australia based on socio-economic disadvantage. English Speaking countries include Canada, Ireland, NZ, South Africa, UK, and USA. Significant differences were determined with a generalized-linear-model (GLM). Means are adjusted for low-energy reporters (energy intake: basal metabolic rate ratio of <0.87). Survey-specific weighting factors were applied. Different letters in columns for each variable indicate significant differences (*p* < 0.05). # Not significant when adjusting for education, age, and income (*p* = 0.09). Values in bold indicate significant trends.
